# Timing of hypothermic temperature control does not affect neurological outcomes after cardiac arrest: a systematic review and meta-analysis

**DOI:** 10.1186/s40635-026-00947-9

**Published:** 2026-07-17

**Authors:** Krisztina Csőke-Kabai, Zsolt Molnár, László Zubek, Dávid Laczkó, Caner Turan, Péter Hegyi, Emőke Henrietta Kovács, Gábor Nagy, Sude Yilmaz, Zoltán Sipos, Krisztián Tánczos

**Affiliations:** 1 Centre for Translational Medicine, Semmelweis University, Budapest, Hungary; 2 Emergency Department, University of Szeged, Szeged, Hungary; 3 Department of Anaesthesiology and Intensive Therapy, Semmelweis University, Budapest, Hungary; 4 Department of Anaesthesiology and Intensive Therapy, Poznan University for Medical Sciences, Poznan, Poland; 5 Department of Interventional Radiology, Heart and Vascular Centre, Semmelweis University, Budapest, Hungary; 6 Institute for Translational Medicine, Medical School, University of Pécs, Pécs, Hungary; 7 Institute of Pancreatic Diseases, Semmelweis University, Budapest, Hungary; 8 Department of Emergency Medicine, Semmelweis University, Budapest, Hungary

**Keywords:** Prehospital, Timing, Hypothermic temperature control, Out-of-hospital cardiac arrest

## Abstract

Out-of-hospital cardiac arrest is associated with a low rate of favourable neurological recovery, estimated at 8%. Lowering the cerebral metabolic rate with hypothermic temperature control is hypothesized to improve this outcome. Given the importance of temperature control after cardiac arrest, we investigated whether initiating early hypothermic temperature control in the prehospital setting could further enhance neurological recovery. A pre-registered systematic search (PROSPERO: CRD42024601051) was run in PubMed, Embase and Cochrane Central on November 3, 2024 (updated on March 30, 2026) to identify randomised controlled trials that compare prehospital vs. in-hospital initiation of hypothermic temperature control of out-of-hospital cardiac arrest patients. The Cochrane Handbook was followed for data extraction and synthesis. The RoB 2 tool was used for risk of bias assessment. The meta-analyses were conducted using a random-effects model. A total of 7117 articles were screened, and 7 studies including 3288 patients were included in the analysis. The intervention group achieved target temperature of 33 °C significantly earlier (MD: -97.27 min; 95% CI: -139.46 to -55.08; *p* = 0.010). In the intervention group, no significant difference was observed in the odds for favourable neurological outcome (OR: 0.98; 95% CI: 0.80–1.22, *p* = 0.855). The rate of recurrent cardiac arrest in the intervention group was significantly higher than in the control group (OR: 1.33; 95% CI: 1.20–1.47; *p* = 0.009) and the odds for pulmonary oedema were also significantly higher (OR: 1.66; 95% CI: 1.24–2.23; *p* = 0.001). The results show that despite reaching the target temperature significantly earlier, prehospital initiation of hypothermic temperature control did not improve favourable neurological recovery compared to in-hospital initiation. Furthermore, early initiation also significantly increased the odds for recurrent cardiac arrest and pulmonary oedema. Therefore, these findings do not support the implementation of prehospital hypothermic temperature control in out-of-hospital cardiac arrest, although evidence regarding fluid-sparing approaches such as transnasal evaporative cooling remains limited.

**Supplementary Information:**

The online version contains supplementary material available at 10.1186/s40635-026-00947-9.

## Introduction

Cardiac arrest (CA) remains a major global public health concern, affecting over 600,000 individuals annually in the United States and Europe, both in out-of-hospital (OHCA) and in-hospital (IHCA) settings [1, 2]. Despite advances in cardiopulmonary resuscitation (CPR) and post-resuscitation care, survival remains low, and neurological disability is common. Among non-traumatic OHCA patients treated by the Emergency Medical Services (EMS), neurologically intact survival is only about 8% [3]. A major cause of morbidity is hypoxic-ischemic brain injury (HIBI), which results from both primary ischemia during arrest and secondary injury after reperfusion [4, 5].

Hypothermic temperature control (HTC) is currently one of the most extensively studied interventions, supported by a substantial body of literature − endorsed by the European Resuscitation Council and the European Society of Intensive Care Medicine (ERC-ESICM), the American Heart Association (AHA) and the International Liaison Committee on Resuscitation (ILCOR) − for mitigating secondary brain injury following CA [6, 7]. Its neuroprotective effects are attributed to reducing cerebral metabolic rate, excitotoxicity, inflammation, and blood-brain barrier disruption, all of which are temperature-dependent [8, 9].

Two landmark trials in the early 2000 s − the Hypothermia After Cardiac Arrest (HACA) study and Bernard et al. − showed that hypothermic temperature control (32–34 °C for 12–24 h) improved neurologic outcomes in comatose OHCA patients with shockable rhythms [10, 11], prompting widespread adoption. However, later trials, including TTM1 and TTM2, showed no significant difference between hypothermia (33 °C) and normothermia (36–37.5 °C) [12, 13]. In TTM2, approximately 80% of patients received bystander CPR − much higher than typical clinical rates (35–40%) − potentially shortening “no-flow” time and limiting the effectiveness of hypothermic temperature control [14]. When evaluating neurological outcomes following out-of-hospital cardiac arrest, a recent meta-analysis identified the HTC score − derived from patients managed with hypothermic temperature control − as the most accurate prognostic tool at 6-month follow-up [15].

Timing may be key to HTC efficacy. Preclinical and observational studies suggest that earlier initiation may better protect the brain, possibly addressing both primary and secondary injury [9, 16]. This has renewed interest in prehospital HTC, particularly intra-arrest or immediate post-ROSC cooling. A prior meta-analysis found no significant benefit, though it paid limited attention to cooling methods [17]. A post hoc analysis of the Continuous Chest Compressions trial linked shorter time to HTC initiation with improved outcomes, reinforcing the compelling hypothesis of early initiation [16].

Considering the evolving and heterogeneous evidence, this meta-analysis aims to assess the impact of early versus later initiation of hypothermic temperature control on neurological outcomes, survival, and safety in OHCA. We further examine the influence of cooling methods and patient subgroups, and discuss limitations of prior studies, ongoing trials, and future directions.

## Methods

This systematic review and meta-analysis was conducted in accordance with the PRISMA 2020 guidelines [18], and adhered to the methodological recommendations of the Cochrane Handbook for Systematic Reviews of Interventions [19]. The study protocol was registered on PROSPERO (registration number: CRD42024601051). The Prisma checklist is included in the Supplementary Material Table S1.

### Information sources

A comprehensive literature search was conducted on November 3, 2024, using the MEDLINE via PubMed, Cochrane Register of Controlled Trials (Central), and Embase databases. Backward and forward citation searching was performed on January 15, 2025. The literature search was then updated on 30 March, 2026 to scan publications in the same three databases that were published since November 4, 2024. The identified articles went through the same selection process and there were no new studies identified or included. The PRISMA flowchart for the updated search is presented in the Supplementary Material on Diagram S1.

### Search strategy

The search key comprised three domains: (1) the intervention procedure (2), the patient population, and (3) the study design, which was limited exclusively to randomized controlled trials (RCTs). The main key used in PubMed was the following: (“hypothermia” OR “cooling” OR (“targeted” AND “temperature” AND “management”) OR (“temperature” AND “control”)) AND (((“cardiopulmonary” OR “cardio-pulmonary”) AND (“arrest” OR “resuscitation”)) OR resuscitat* OR (“cardiac” AND “arrest”) OR “ALS” OR (“advanced” AND “life” AND “support”) OR (“return” AND “spontaneous” AND “circulation”) OR “ROSC”) AND (random* OR blind* OR controlled clinical trial [pt] OR randomized controlled trial [pt]). The additional search keys tailored to each database are provided in the Supplementary Material Table S2. Aside from filtering by study type, no restrictions were applied regarding language or other parameters.

### Eligibility criteria

We included randomized controlled trials published in peer-reviewed scientific journals that investigated patients who experienced out-of-hospital cardiac arrest (OHCA). Our PICO framework was the following: The study population consisted of adult patients of both sexes who experienced out-of-hospital cardiac arrest, were resuscitated by EMS, and subsequently transported to hospital. The aetiology of cardiac arrest and the initial cardiac rhythm were heterogeneous, with the exception of traumatic causes, and patients were comatosed and mechanically ventilated at the time of enrolment. The intervention was initiation of HTC in the prehospital setting with continuation after hospital admission, compared with HTC initiated exclusively in-hospital. The primary outcome was favourable neurological function at hospital discharge. Secondary outcomes included: survival to hospital discharge, 90-day mortality, recurrent arrest, body temperature at hospital admission, time to achieve target temperature, and adverse events such as arrhythmias, pulmonary oedema, acidosis, vasopressor requirement after return of spontaneous circulation (ROSC), and bleeding. Exclusion criteria included paediatric population (below 12 years), trauma patients, studies in which prehospital HTC was discontinued after hospital admission, non-peer reviewed studies, and studies without full text available.

### Selection process

Study selection was managed using Endnote citation manager software [20]. Two independent reviewer teams (KCK with the help of SY, and GN separately) screened titles and abstracts following duplicate removal, then assessed full texts for eligibility. The two separate lists of studies to be included were then compared, to ensure rigorous methodology. The Cohen’s kappa was 0.81 for title and abstract selection and 0.86 for full-text selection. Discrepancies at any stage of selection were resolved through discussion with a third independent reviewer (DL). The literature search update was performed by the first author.

### Data collection process

Data were extracted by two independent investigators (KCK and GN) into a standardized data table which was specifically tailored for this project by the first author. The extracted data tables were cross-validated by the first author to identify and rectify discrepancies.

### Data items

Extracted variables included: first author, year of publication, study location, study time interval, study population, demographic characteristics, and initial cardiac rhythm. Intervention details comprised: number of patients, cooling initiation time, method of cooling, target temperature, and time from collapse to advanced life support (ALS) team arrival for both intervention and control groups. On separate Excel sheets, the authors also extracted the variables needed to calculate differences in the secondary outcomes and adverse events listed above.

### Definition of the primary outcome

The primary outcome was favourable neurological outcome at hospital discharge, and it was defined as the ability to live independently, reported either explicitly or as Cerebral Performance Category (CPC) scores of 1 or 2. Studies not employing CPC used equivalent criteria aligned with independent living, allowing consistent aggregation under the term favourable neurological outcome. Some studies defined favourable neurological outcome outcome as the patient being discharged home or being eligible for rehabilitation. The study by Nordberg et al. reported neurological outcomes at 90 days, which we deemed comparable and thus poolable with discharge-time assessments [21].

### Study risk of bias and certainty of evidence assessment

Two authors (KCK and GN) independently assessed the risk of bias using the RoB-2 tool [22] for randomised trials, and discrepancies were resolved by a third independent reviewer (DL). We evaluated the certainty of evidence using the GRADE (Grading of Recommendations Assessment, Development and Evaluation) approach [23].

### Synthesis methods

A meta-analysis was performed for the pre-defined outcomes. Conventional meta-analyses were carried out by using the MetaBoostR software; a modular, automated R framework for meta-analysis (from R Core Team, 2025) [24]. For dichotomous outcomes, we calculated odds ratios (ORs) with 95% confidence intervals (CIs); for continuous outcomes, mean differences (MDs) with 95% CIs were calculated. The random effects model was used. Forest plots were used to visually present individual and pooled study outcomes. Publication bias could not be assessed by visual inspection of the Funnel plot or by Egger’s test due to the small number of available studies.

Studies that fit our framework but did not present data appropriately were only included for qualitative synthesis. Heterogeneity was assessed using the I² statistic and Chi-squared (χ²) test, with *p* < 0.1 considered indicative of significant heterogeneity. Statistical significance for all other analyses was set at *p* < 0.05.

### Subgroup analyses

Subgroup analyses were conducted for the primary outcome (favourable neurological status) stratified by initial rhythm, EMS response time after collapse (< 10 vs. ≥10 min), method of cooling, and timing of cooling initiation (intra-arrest vs. post-ROSC). Subgroup analyses for survival outcomes were also stratified by initial rhythm.

## Results

### Search and selection

**Fig. 1:**
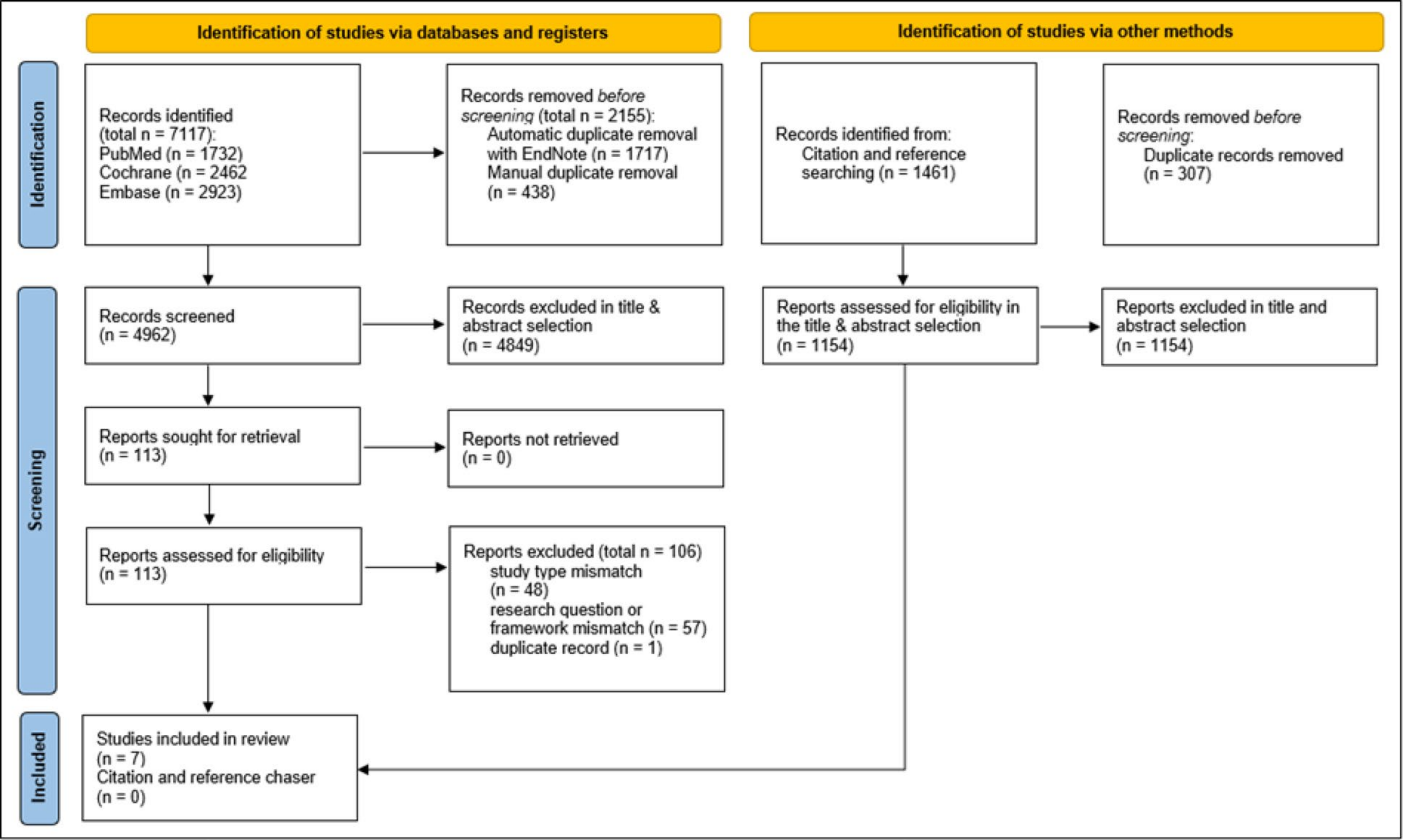
PRISMA flowchart of selection of studies

A total of 7117 studies were identified using the search key. After the removal of duplicates, 4962 articles were screened based on title and abstract. Ultimately, 7 full-text articles [25–31] were deemed eligible for inclusion from among the 113 full-text studies assessed (as shown in Fig. 1).

### Baseline characteristics of included studies

Baseline characteristics of the included RCTs are detailed in Table 1. with extended data table available in Supplementary material Table S3.

**Table 1:** Baseline characteristics of the included studies

Study	Population	Initial rhythm	Method of cooling in intervention group	Start of cooling on the prehospital scene	Control group	Target T (°C)	Definition of favourable neurological outcome in the individual study
Bernard et al., 2010	Adults with OHCA, VF	Ventricular fibrillation (VF)	Rapid IV infusion	Post-ROSC	Intrahospital cooling	33	Discharge to home or rehabilitation
Bernard et al., 2012	OHCA, asystole or PEA	Asystole or pulseless electrical activity (PEA)	Rapid IV infusion	Post-ROSC	Intrahospital cooling	32–34	Discharge to home or rehabilitation
Bernard et al., 2016	OHCA	All rhythms	Rapid IV infusion	Intra-arrest	Intrahospital cooling	33	Discharge to home or rehabilitation
Castrén et al., 2010	OHCA	All rhythms	Transnasal evaporative device	Intra-arrest	Intrahospital cooling	34	CPC 1–2 at hospital discharge
Debaty et al., 2014	OHCA	All rhythms	IV infusion + gel pads	Intra-arrest	Intrahospital cooling	32–34	CPC 1–2 at hospital discharge
Kim et al., 2014	OHCA	VF	IV infusion of 4 °C saline	Post-ROSC	Intrahospital cooling of VF patients*	< 34 °C	Full recovery or mild impairment at discharge
Nordberg et al., 2019	OHCA	All rhythms	Transnasal evaporative device	Intra-arrest	Intrahospital cooling	32–34	CPC 1–2 at 90 days

*selective intervention: only VF patients were cooled in the hospital

### Time to attainment of target temperature

**Fig. 2:**
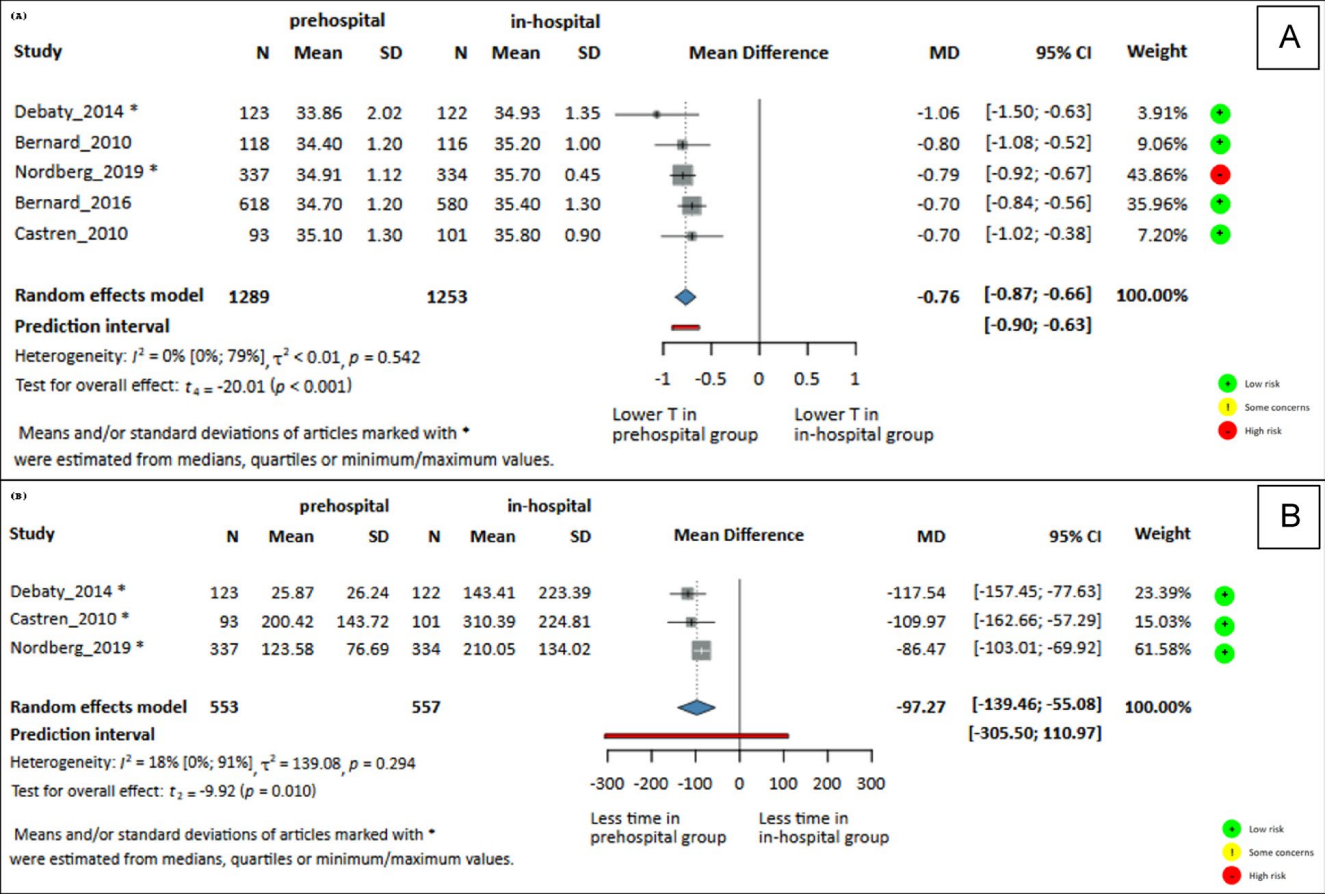
Effectiveness of the procedure. (**A**) Forest plot of core temperature (°C) at hospital arrival showing that patients with prehospital hypothermic temperature control had a significantly lower body temperature upon arrival. N=number of patients; T=temperature; SD=standard deviation; MD=mean difference; CI=confidence interval. (**B**) Forest plot showing significantly shorter mean difference in time to reach the target temperature of 33 °C in patients with prehospital hypothermic temperature control. Time presented in minutes. N=number of patients; MD=mean difference; SD=standard deviation; CI=confidence interval

A statistically significant difference was observed in the body temperature of patients upon hospital admission (Fig. 2., plot (A)). Patients with prehospital-initiated cooling exhibited significantly lower body temperatures upon hospital admission compared to those who received only in-hospital temperature control (MD: −0.76 °C; 95% CI: −0.87 to −0.66; *p* < 0.001). Furthermore, these patients reached the target temperature of 33 °C significantly earlier, by approximately one and a half hours, regardless of the cooling method employed (MD: −97.27 min; 95% CI: −139.46 to −55.08 min; *p* = 0.010) (Fig. 2., plot (B)).

### Primary outcome: favourable neurological recovery

**Fig. 3:**
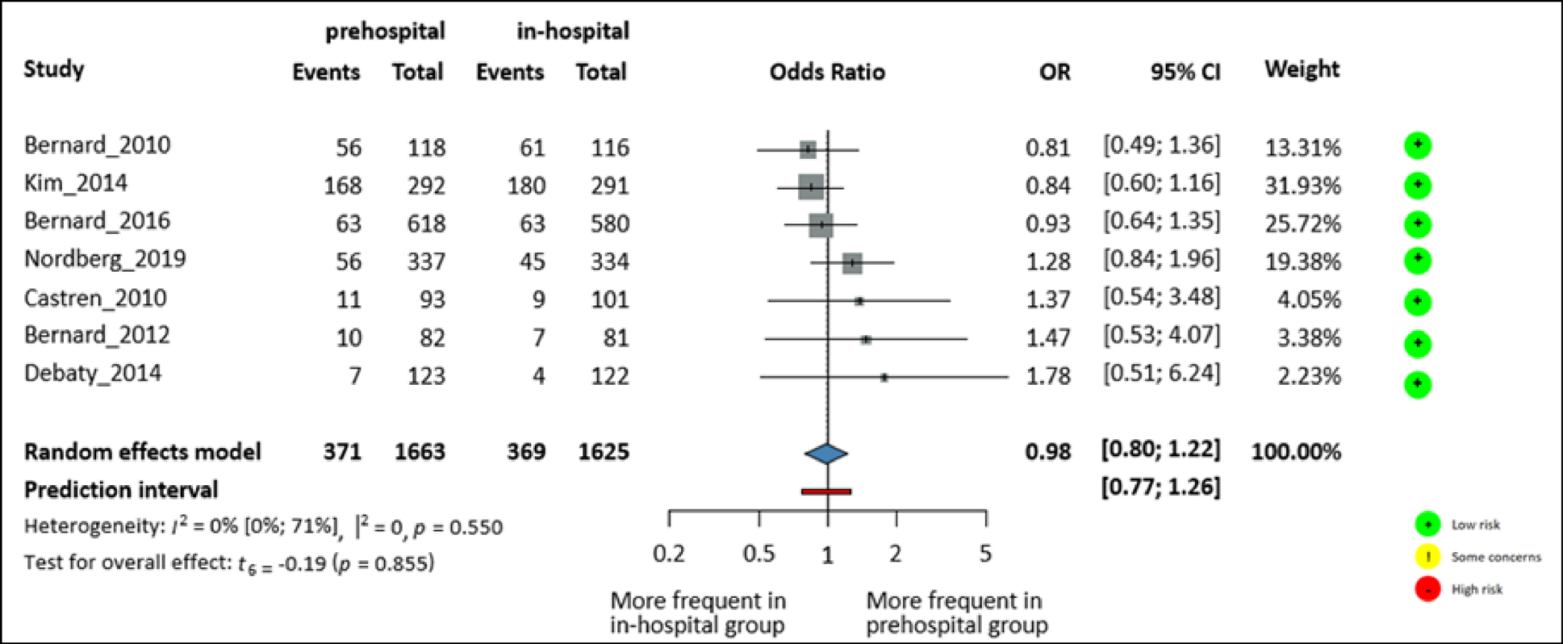
Odds for favourable neurological recovery. Forest plot showing a non-significant difference in the odds of favourable neurological outcome of patients at hospital discharge in the intervention and control group. OR=odds ratio; CI=confidence interval

No statistically significant differences were observed in neurological outcomes between patients who received early, prehospital-initiated temperature control and those whose cooling was started in-hospital (OR: 0.98; 95% CI: 0.80–1.22; *p* = 0.855) (Fig. 3). Subgroup analyses based on the predefined factors consistently showed no neurological benefit in patients with shockable (VF) versus non-shockable rhythms (OR: 1.06, 95% CI: 0.54–2.08, *p* = 0.785 vs. OR: 1.06, 95% CI: 0.02–51.36, *p* = 0.883); those who received professional ALS within versus over 10 min of collapse (OR: 1.04, 95% CI: 0.60–1.79, *p* = 0.867 vs. OR: 1.32, 95% CI: 0.65–2.66, *p* = 0.297); those who underwent different modes of temperature control (intravenous cold fluids [OR: 0.90, 95% CI: 0.72–1.14, *p =* 0.299] or intranasal method [OR: 1.30, 95% CI: 0.93–1.80, *p* = 0.064); and those whose prehospital HTC was initiated intra-arrest (OR: 1.11 95% CI: 0.78–1.58, *p* = 0.400) or post-ROSC (OR: 0.86 95% CI: 0.55–1.34, *p* = 0.288) from earlier onset of the procedure (forest plots of mentioned subgroups are presented in the Supplementary Material on Figures S1-S8).

### Secondary outcomes

In regards to survival to discharge, patients whose cooling begun prehospitally did not have higher odds of survival compared to the control group (OR: 0.98, 95% CI: 0.83–1.17; *p* = 0.839), and this was also the trend when examining survival in patients with shockable and non-shockable initial rhythm (OR: 0.94, 95% CI: 0.69–1.28; *p* = 0.595 and (OR: 1.27, 95% CI: 0.39–4.08; *p* = 0.474). Forest plots of mentioned analyses are presented in the Supplementary Material on Figures S9-S11. 90-day mortality and the changes in pH levels were pre-defined secondary outcomes, although due to insufficient available data, those calculations were not performed.

### Safety outcomes

**Fig. 4:**
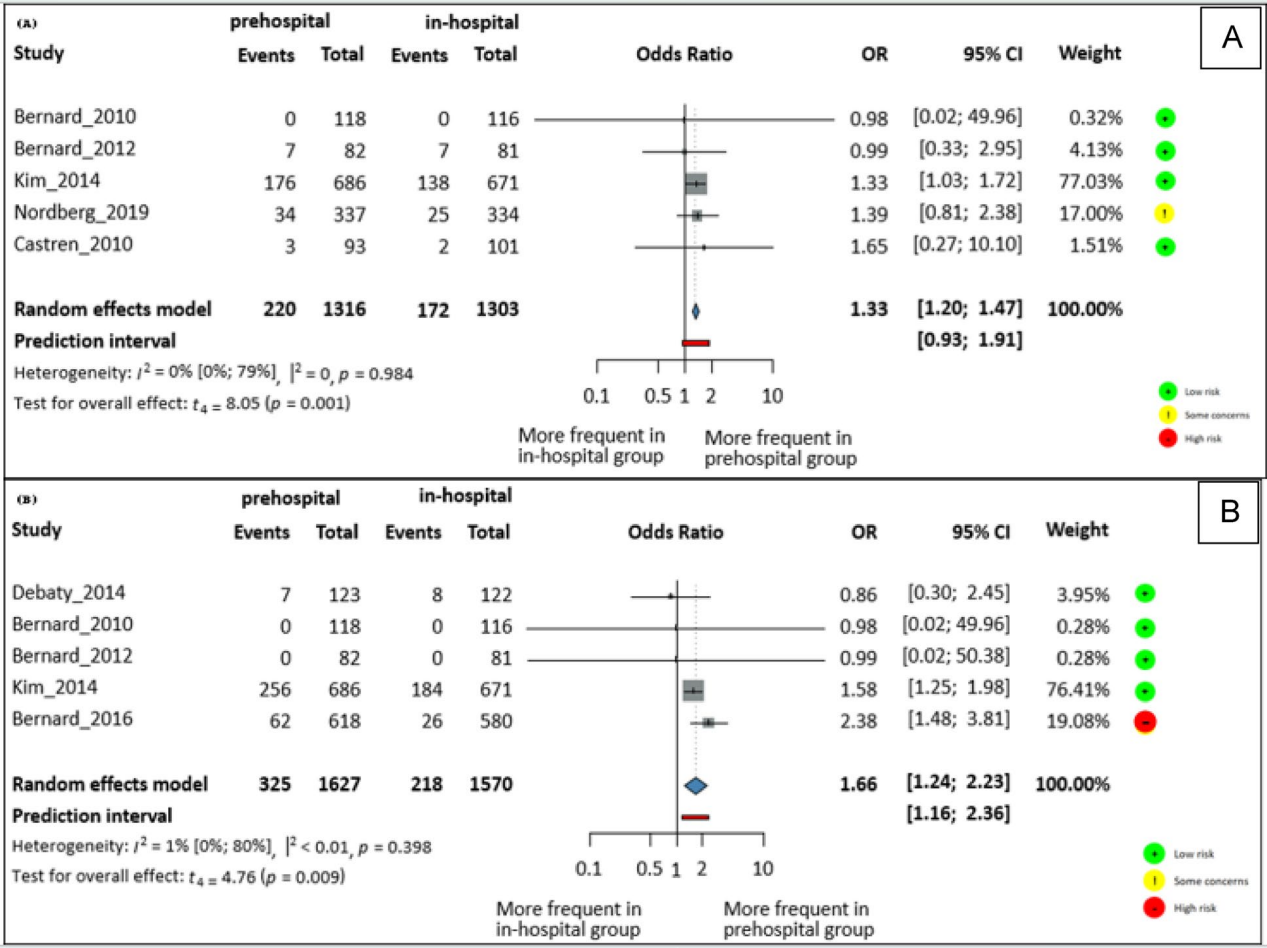
Safety concerns of early-initiated temperature control. (**A**) Forest plot showing significantly increased odds for recurrent cardiac arrest in the prehospital group irrespective of method of cooling. OR=odds ratio; CI=confidence interval. (**B**) Forest plot showing significantly increased odds for pulmonary oedema in the prehospital group. All included studies utilized large-volume cold intravenous fluid for cooling, the overall median (Q1-Q3) in the five studies is 1625 mL (1375, 1787.5 mL). OR=odds ratio; CI=confidence interval

Regardless of the cooling method, patients in the prehospital cohort exhibited significantly increased odds of recurrent cardiac arrest (RA) (OR: 1.33; 95% CI: 1.20–1.47; *p* = 0.001) (Fig. 4., plot (A)). Additionally, the analysis revealed that patients in the early cooling group had significantly higher odds of developing pulmonary oedema when the cooling protocol involved large volumes of intravenous fluids (OR: 1.66; 95% CI: 1.24–2.23; *p* = 0.009) (Fig. 4., plot (B)).

**Fig. 5:**
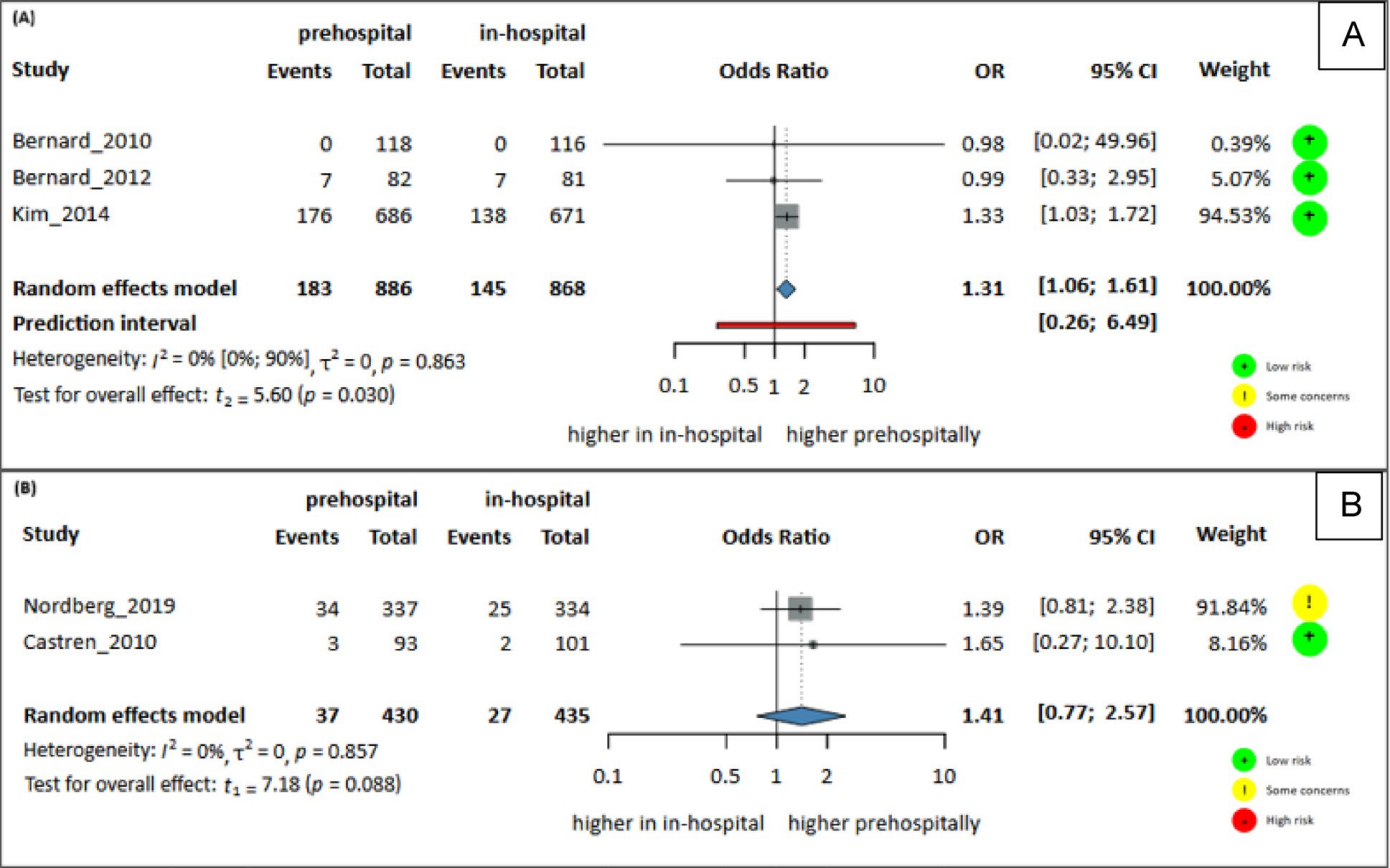
Difference in the rate of recurrent cardiac arrests regarding cooling method. (**A**) Forest plot showing significantly elevated rate of recurrent cardiac arrests in patients with prehospital hypothermic temperature control by large volumes of intravenous fluids. OR=odds ratio; CI=confidence interval. (**B**) Forest plot showing a non-significant difference in the rate of recurrent cardiac arrests in patients with prehospital hypothermic temperature control by transnasal evaporative device. OR=odds ratio; CI=confidence interval

Subgroup analysis by cooling methods revealed a significant increase in recurrent cardiac arrest in the intravenous cooling group (OR: 1.31; 95% CI: 1.06–1.61; *p* = 0.030), whereas the transnasal group showed only a non-significant difference (OR: 1.41; 95% CI: 0.77–2.57; *p* = 0.088) (Fig. 5).

Additional analyses of safety outcomes such as vasopressor requirement (OR: 0.95; 95% CI: 0.50–1.80; *p* = 0.755), major bleeding (OR: 1.58; 95% CI: 0.78–3.20; *p* = 0.107), and arrhythmias (OR: 0.91; 95% CI: 0.40–1.39; *p* = 0.456) did not reveal statistically significant differences between the intervention and control group. The forest plots of those analyses are presented in the Supplementary Material on Figures S12-S14.

### Risk of bias and GRADE assessment

The results of the risk of bias assessment are presented in the forest plots within this manuscript, with most outcomes across studies rated at low risk. However, specific concerns were noted for some secondary outcomes. Overall, the certainty of evidence was moderate to high across outcomes according to the GRADE framework. Evidence for neurological outcomes, survival, temperature at hospital admission, and time to target temperature was rated as high certainty, whereas pulmonary oedema, recurrent cardiac arrest, and vasopressor requirement were downgraded to moderate certainty mainly because of either incomplete outcome reporting, or heterogeneity in outcome assessment timing, or inconsistencies in outcome definitions across studies. Detailed risk of bias and GRADE assessments are provided in the Supplementary Material on Figures S15-S23 and in Table S4.

## Discussion

This meta-analysis examined whether early initiation of hypothermic temperature control improves neurological outcomes compared with delayed initiation. Our study demonstrates that although early-initiated HTC significantly accelerates the attainment of the target temperature of 33 °C, this earlier start does not translate into improved neurological outcomes in OHCA. Notably, prehospital cooling is associated with a higher risk of recurrent cardiac arrest and pulmonary oedema, raising substantial safety concerns regarding its use in the prehospital setting. These findings align with the latest 2025 ERC-ESICM Guidelines on post-resuscitation care, further reinforcing the recommendation against implementing prehospital cooling using large volumes of intravenous fluids [6]. While the guidelines reference a single randomized controlled trial indicating the potential harms of this approach, the present meta-analysis contributes novel, higher-level evidence that strengthens the certainty of this conclusion.

Hypoxic-ischemic brain injury is a major cause of morbidity after ROSC, driven by both the initial insult and secondary mechanisms lasting hours to days [14]. These processes are highly temperature-sensitive; even brief post-ROSC hyperthermia (> 37.7 °C) worsens outcomes [32]. A single-centre study reviewing post-cardiac arrest syndrome underscored the importance of hypothermic temperature control, describing its physiological effects and limitations in improving outcomes [33].

In our meta-analysis, prehospital-initiated hypothermic temperature control resulted in significantly lower core temperatures upon hospital arrival and significantly faster achievement of the target temperature − by saving approximately 1.5 h. Despite the apparent clinical efficacy of the procedure to cool down OHCA patients, still no statistically significant benefit was observed in terms of favourable neurological recovery, the primary outcome of this study. Furthermore, the absence of neurological benefit was consistent across multiple predefined subgroups. This uniformity across subgroups strengthens the conclusion that earlier induction of hypothermic temperature control does not confer neurological advantage over delayed in-hospital initiation. A recently published meta-analysis, which included a more heterogeneous population comprising both OHCA and in-hospital cardiac arrest (IHCA) patients, hypothesized that achieving the target temperature early − within 30 min of return of spontaneous circulation (ROSC), when reactive oxygen species (ROS) production peaks − would improve survival with favourable neurological outcomes [34]. The authors proposed that failure to achieve target temperature within this therapeutic window may have contributed to the observed lack of improvement in neurological outcomes.

Our findings challenge the intuitive assumption that “earlier is better” when it comes to hypothermic temperature control after cardiac arrest. While preclinical models [35, 36] and small-scale studies [16] have suggested that early initiation of cooling may attenuate cerebral ischemia-reperfusion injury, the clinical reality appears to be more complex. Several factors may explain the lack of benefit observed. First, systemic hypothermia is only one of several variables influencing neurological recovery; its effect may be insufficient to overcome the injury already sustained during the cardiac arrest. Second, the quality and timing of CPR, reperfusion strategies, and post-resuscitation care likely exert a greater impact on outcomes than the marginal time difference in cooling observed here [14].

Importantly, our results reveal significant safety concerns associated with prehospital HTC, particularly when large volumes of cold intravenous fluids are administered. The incidence of recurrent cardiac arrest was significantly higher in patients receiving early cooling. Furthermore, pulmonary oedema occurred more frequently with prehospital HTC in the intravenous cooling subgroup, underscoring the hemodynamic and pulmonary risks of fluid-based cooling methods. These adverse events likely stem from fluid overload, hemodynamic changes affecting preload and afterload, and the cooling-induced peripheral vasoconstriction that can impair cardiac function in the immediate post-arrest period [14].

Our subgroup analysis by cooling method further supports this concern. Even though IV and transnasal temperature control was not directly compared to each other, the analysis shows that the transnasal method might not pose such dangers. Intravenous fluid cooling was significantly associated with higher rates of recurrent cardiac arrest, while transnasal cooling was not associated with a statistically significant effect on this outcome, though interpretation is limited by the small number of studies (*n* = 2). Unlike intravenous cold fluids, the transnasal device delivers non-invasive cooling via cooled oxygen, without fluid loading. This distinction is clinically relevant, as excessive fluid administration may contribute to hemodynamic instability and pulmonary complications.

Although our meta-analysis of pooled RCT data found no significant difference in neurological outcomes between intravenous and transnasal cooling methods, it is noteworthy that an individual participant data meta-analysis reported improved neurological recovery with transnasal cooling in patients presenting with initial ventricular fibrillation [37]. This is consistent with findings from Castrén et al., who observed significantly better neurological outcomes in patients receiving professional CPR and transnasal evaporative cooling within 10 min of collapse compared to in-hospital HTC alone [29].

These findings suggest that fluid-sparing approaches, such as transnasal cooling, may offer potential clinical benefits with fewer adverse events in the prehospital setting. However, due to heterogeneity in methodologies and limited comparative data, further studies are warranted to determine the relative safety and efficacy of fluid-sparing cooling strategies that also focus on the importance of the early therapeutic window. Notably, a randomized trial investigating prehospital-initiated ultrafast HTC using transnasal evaporative device in OHCA patients with initial shockable rhythm is currently ongoing [38].

### Strengths and limitations

This meta-analysis has several strengths, including following our pre-registered rigorous protocol, a large pooled sample size, different subgroup and safety analyses, and the use of high-quality randomized studies. However, limitations must also be acknowledged. Heterogeneity in aetiology, cooling protocols and methods, EMS systems, transport times across studies may have introduced variability that could dilute potential effects. The included trials generally reported major prehospital prognostic variables, including bystander CPR and arrest-time intervals, and these were mostly balanced between randomized groups. However, reporting of downstream postarrest care was less consistent. Hemodynamic management, sedation, neuroprognostication, and detailed in-hospital temperature-control practices were either protocol-driven but not reported by group or incompletely described. Therefore, these factors could not be formally explored in subgroup or meta-regression analyses and may have contributed to residual clinical heterogeneity. Moreover, the analysis of some outcomes (especially in the case of transnasal cooling) was limited by the small number of available studies. Regarding times of outcome assessment, the study form Nordberg et al. analysed neurological outcomes at 90 days as opposed to the other included ones assessing at hospital discharge.

### Implication for practice and research

Our results suggest that prehospital cooling should be avoided due to increased risks of pulmonary oedema and recurrent cardiac arrest without clear neurological benefit. Transnasal cooling was not associated with significantly higher adverse events, although available evidence is limited. Further studies are needed to clarify its safety and potential efficacy. To ensure such evidence translates into safer and more effective patient care, robust translational medicine frameworks are needed to bridge research findings with routine clinical practice [39, 40].

## Conclusion

While early cooling in the prehospital setting achieves target temperature more rapidly, it does not improve neurological outcomes and may increase the risk of adverse events, particularly when using intravenous fluid-based methods. Fluid-sparing cooling strategies, such as transnasal cooling devices should be further studied.

### Take-home message

Early initiation of hypothermic temperature control resulted in significantly earlier achievement of target temperature but was not associated with improved favourable neurological outcomes compared with later, in-hospital initiation. In contrast, it was associated with increased odds of recurrent cardiac arrest and pulmonary oedema.

### Tweet

Early timing of hypothermic temperature control after OHCA achieves target temperature earlier, but shows no neurological benefit compared to later initiation, while increasing adverse events.

## Supplementary Information

Supplementary Material 1

## Data Availability

None of the analysed data that we used is original data, the original published data can be found in the cited articles.
