# Supplementary material for: Timing of hypothermic temperature control does not affect neurological outcomes after cardiac arrest: a systematic review and meta-analysis
Source: Intensive Care Med Exp. 2026 Jul 17;14:96. doi: 10.1186/s40635-026-00947-9 (PMC13379534; doi:10.1186/s40635-026-00947-9)
Supplement: Supplementary file 1 — Supplementary Material 1 [file 40635_2026_947_MOESM1_ESM.docx]

**Timing of Hypothermic Temperature Control Does Not Affect Neurological Outcomes After Cardiac Arrest: A systematic review and meta-analysis**

**SUPPLEMENTARY MATERIAL**

**Authors**

Krisztina Csőke-Kabai ^a, b^, Zsolt Molnár ^a, c, d^, László Zubek ^a, c^, Dávid Laczkó ^a, e^, Caner Turan ^a, c^, Péter Hegyi ^a, f, g^, Emőke Henrietta Kovács ^a^, Gábor Nagy ^a, h^, Sude Yilmaz ^a^, Zoltán Sipos ^a,^ Krisztián Tánczos ^a, c^

**Affiliations**

1. Centre for Translational Medicine, Semmelweis University, Budapest, Hungary
2. Emergency Department, University of Szeged, Szeged, Hungary
3. Department of Anaesthesiology and Intensive Therapy, Semmelweis University, Budapest, Hungary
4. Department of Anaesthesiology and Intensive Therapy, Poznan University for Medical Sciences, Poznan, Poland
5. Department of Interventional Radiology, Heart and Vascular Centre, Semmelweis University, Budapest, Hungary
6. Institute for Translational Medicine, Medical School, University of Pécs, Pécs, Hungary
7. Institute of Pancreatic Diseases, Semmelweis University, Budapest, Hungary
8. Department of Emergency Medicine, Semmelweis University, Budapest, Hungary

**Corresponding author**

Krisztián Tánczos, MD, PhD

Postal address: Semmelweis University, 1428 Budapest, PO box 2.

Tel.: +36703398119

E-mail address: tanczos.krisztian@semmelweis.hu

**SEARCH STRATEGY AND SEARCH UPDATE**

Prepared by: Krisztina Csőke-Kabai

Date of search: 03-Nov-2024

Title: Investigating the efficacy and safety of prehospital targeted temperature management* on neurological outcome in out-of-hospital cardiac arrest patients

Clinical question: Does early (prehospitally) initiated TTM improve neurological outcome compared to later (in-hospital) started TTM in OHCA patients?

Framework:

P: patients after OHCA

I: TTM started prehospitally

C: TTM started in-hospital

O1: neurological functions

O2: survival to discharge, 90-day mortality, recurrent arrest, body temperature at hospital

admission, time to reach target temperature, adverse events (arrhythmias, pulmonary oedema,

acidosis, vasopressor need after return of spontaneous circulation, bleeding)

(*Please note that the terminology changed in 2025 and now the investigated procedure is called hypothermic temperature control.)

**Search update**

To ensure up-to-date data pool, we repeated the literature search using the same strategy in all three databases with the original search key applying identical settings. The search run was on March 30, 2026 and aimed articles published between November 4, 2024 and the date of the search. In Embase, there were no settings available for the exact date of publishing, therefore we included articles from 2024, 2025 and 2026. Following duplicate removal, the found articles went through title and abstract selection and then full-text selection. We confirm that no new studies were identified or included in the final analysis.


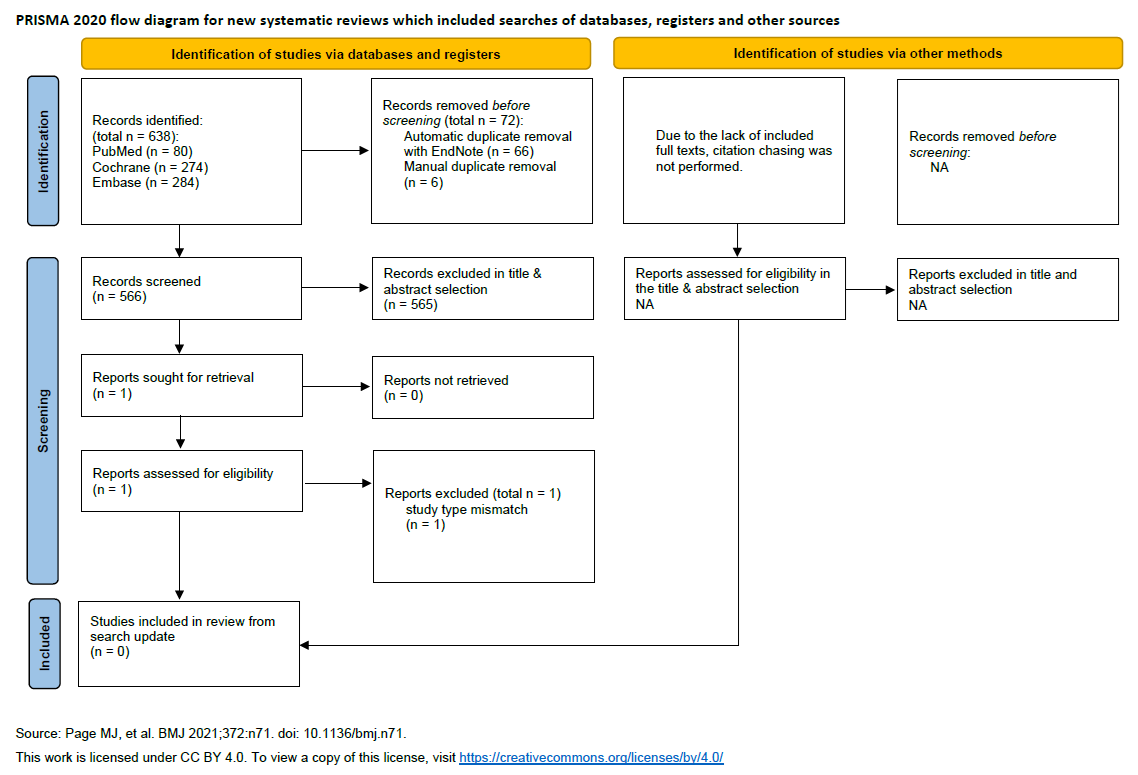


Diagram S1: PRISMA flowchart of the search for articles published between November 4, 2024 (date of previous search) and March 30, 2026

**Prisma checklist**

**
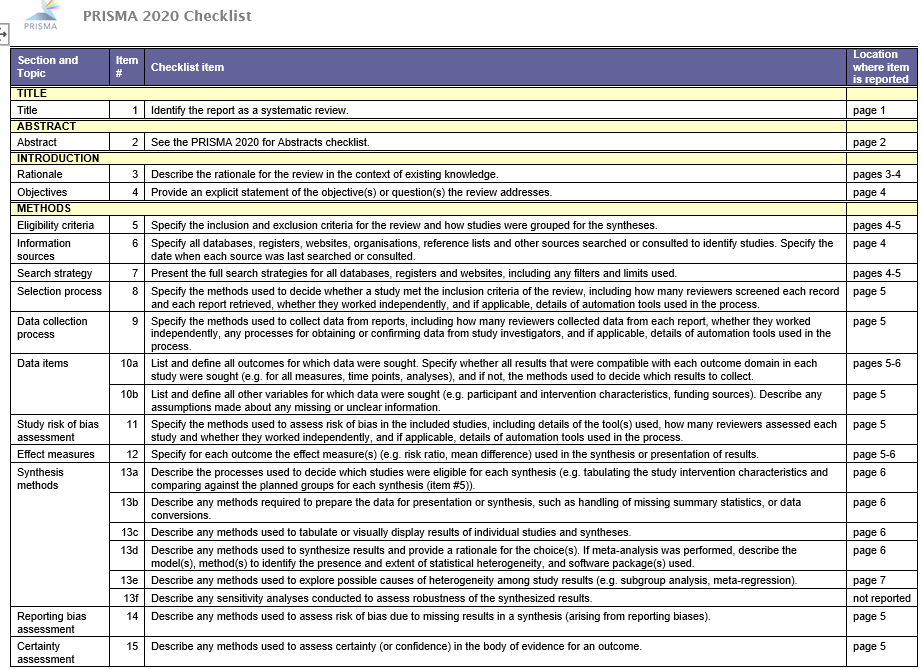
**

**
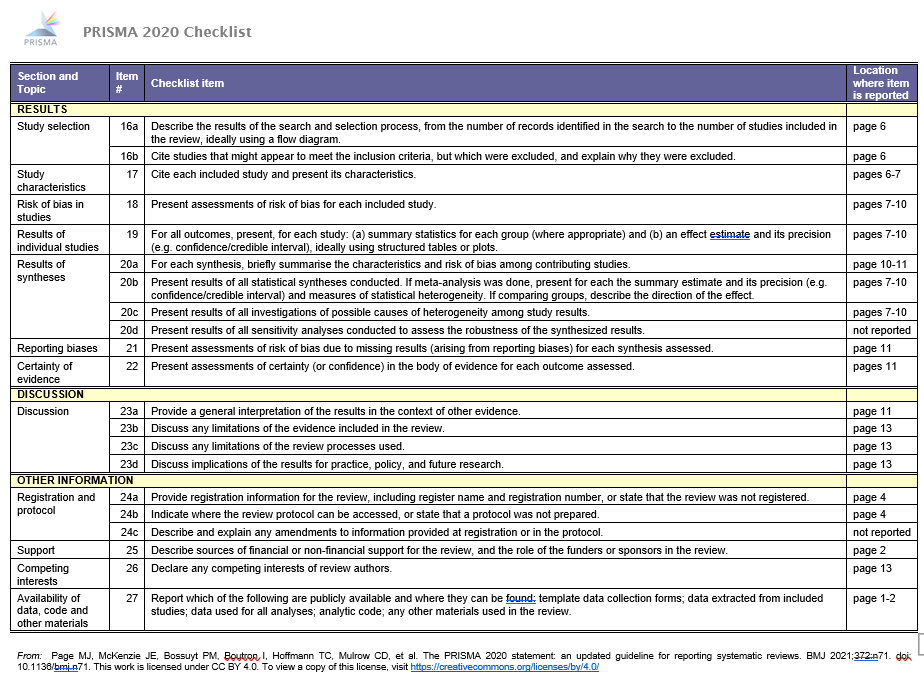
**

Table S1. Prisma checklist

**SEARCHKEYS**

| **DATABASE** | **SEARCH KEYS** | **NOTES** |
| --- | --- | --- |
| **PubMed** | ("hypothermia" OR "cooling" OR ("targeted" AND "temperature" AND "management") OR  ("temperature" AND "control"))  AND  (((”cardiopulmonary” OR ”cardio-pulmonary”) AND (”arrest” OR ”resuscitation”)) OR  resuscitat* OR ("cardiac" AND "arrest") OR "ALS" OR ("advanced” AND ”life”  AND ”support") OR ("return” AND ”spontaneous” AND ”circulation") OR "ROSC")  AND  (random* OR blind* OR controlled clinical trial [pt] OR randomized controlled trial [pt]) | Domain 1 refers to: the intervention, which is targeted temperature management  Domain 2 refers to: resuscitated OHCA patients  Domain 3 refers to: study type Number of results: 1732  Notes: Resuscitat* is for resuscitation or resuscitated. Random* is for  randomization/randomisation or randomized/randomised. Blind* is for blinded or blinding.  Abbreviations are included in the search key because, due to the word count limit, many  abstracts use them.  Database settings: Advanced search was used, all text was searched. |
| **Embase** | (hypothermia OR cooling OR (targeted AND temperature AND management) OR (temperature  AND control))  AND  (((cardiopulmonary OR cardio-pulmonary) AND (arrest OR resuscitation)) OR resuscitat* OR  (cardiac AND arrest) OR ALS OR (advanced AND life AND support) OR (return AND  spontaneous AND circulation) OR ROSC)  AND  (random* OR blind* OR 'randomized controlled trial'/exp OR 'controlled clinical trial'/de) | Domain 1 refers to: the intervention, which is targeted temperature management  Domain 2 refers to: resuscitated OHCA patients  Domain 3 refers to: study type  Number of results: 2923  Notes: Resuscitat* is for resuscitation or resuscitated. Random* is for  randomization/randomisation or randomized/randomised. Blind* is for blinded or blinding.  Abbreviations are in the key because due to the wordcount limit many abstracts use them.  Database settings: Advanced search was used. All checkmarks were turned off. All Text was  searched. The number of hits did not make using :*ab,kw,ti* necessary |
| **CENTRAL (Cochrane)** | (hypothermia OR cooling OR (targeted AND temperature AND management) OR (temperature  AND control))  AND  (((cardiopulmonary OR cardio-pulmonary) AND (arrest OR resuscitation)) OR resuscitat* OR  (cardiac AND arrest) OR ALS OR (advanced AND life AND support) OR (return AND  spontaneous AND circulation) OR ROSC) | Domain 1 refers to: the intervention, which is targeted temperature management  Domain 2 refers to: resuscitated OHCA patients  Number of results: 2462  Notes: Resuscitat* is for resuscitation or resuscitated. Abbreviations are in the key because due  to the wordcount limit many abstracts use them. The parentheses in the first domain were  required by the database. There is no Domain3 here. The results are all trials.  Database settings: Advanced search was used. Only trials are included. All Text was searched. |

Table S2: Search key used in each databases

**BASELINE CHARACTERISTICS** (EXTENDED VERSION)

| **First Author and Year of Publication** | **Bernard et al., 2010** | **Bernard et al., 2012** | **Bernard et al., 2016** | **Castrén et al., 2010** | **Debaty et al., 2014** | **Kim et al., 2014** | **Nordberg et al., 2019** |
| --- | --- | --- | --- | --- | --- | --- | --- |
| **Study Time Interval** | Oct 2005 Nov 2007 | Oct 2005 Nov 2007 | Dec 2010 Dec 2014 | Nov 2008 Jun 2009 | Sep 2009 Jul 2012 | Dec 2007 Dec 2012 | Jan 2010 Jan 2018 |
| **Study Location (Country)** | Australia | Australia | Australia | 5 Eu countries | France | USA | 7 Eu countries |
| **Mean Age (Intervention)** | 63,4 | 63,8 | 65,3 | 66,1 | n/a | 62,1 | 64 |
| **Mean Age (Control)** | 63 | 61,1 | 64,3 | 64,2 | n/a | 62,1 | 66 |
| **Sex, male% (Intervention)** | 83,1 | 69 | 74,8 | 72 | 72,4 | 78 | 75,3 |
| **Sex, male% (Control)** | 86,2 | 59 | 73,8 | 78,2 | 70,5 | 75 | 75,7 |
| **No. of Patients Total** | 234 | 163 | 1198 | 194 | 245 | 583 | 677 |
| **No. of Patients received intervention** | 118 | 82 | 618 | 93 | 123 | 292 | 343 |
| **No of patients in Control Group** | 116 | 81 | 580 | 101 | 122 | 291 | 334 |
| **Study Population** | OHCA | OHCA | OHCA | OHCA | OHCA | OHCA | OHCA |
| **Initial Rhythm** | Ventricular fibrillation (VF) | Asystole or pulseless electrical activity (PEA) | All rhythms | All rhythms | All rhythms | VF | All rhythms |
| **Method of Cooling In Intervention Group** | Rapid IV infusion | Rapid IV infusion | Rapid IV infusion | Transnasal evaporative device | IV infusion + gel pads | IV infusion of 4°C saline | Transnasal evaporative device |
| **Start of Cooling on the Prehospital Scene** | Post-ROSC | Post-ROSC | Intra-arrest | Intra-arrest | Intra-arrest | Post-ROSC | Intra-arrest |
| **Control Group** | Intrahospital cooling | Intrahospital cooling | Intrahospital cooling | Intrahospital cooling | Intrahospital cooling | Intrahospital cooling of VF patients* | Intrahospital cooling |
| **Target T (**°C) | 33 | 32–34 | 33 | 34 | 32–34 | <34°C | 32–34 |
| **Definition of Favourable Neurology and Follow-up Time** | Hospital discharge to home or rehabilitation | Hospital discharge to home or rehabilitation | Hospital discharge to home or rehabilitation | CPC 1–2 at hospital discharge | CPC 1-2 at hospital discharge | Full recovery or mild impairment at discharge | CPC 1–2 at 90 days |
| **Time from collapse to ALS team (mean**±**SD) Intervention** | 8.4±3.1 | 13.1±6.3 | 9.2±4.2 | 12.3** | 17.** | 5.3±2 | 9** |
| **Time from collapse to ALS team (mean** ±**SD) Control** | 8.9±3.3 | 13.5±5.4 | 8.8±3.7 | 11.33* | 18.5** | 5.2±2.1 | 9.667** |

*: selective intervention: only VF patients were cooled in the hospital

**: the mean numbers were calculated from median (IQ1. IQ3).

Table S3: Baseline characteristics of included articles, extended version

**PRIMARY OUTCOME**

*
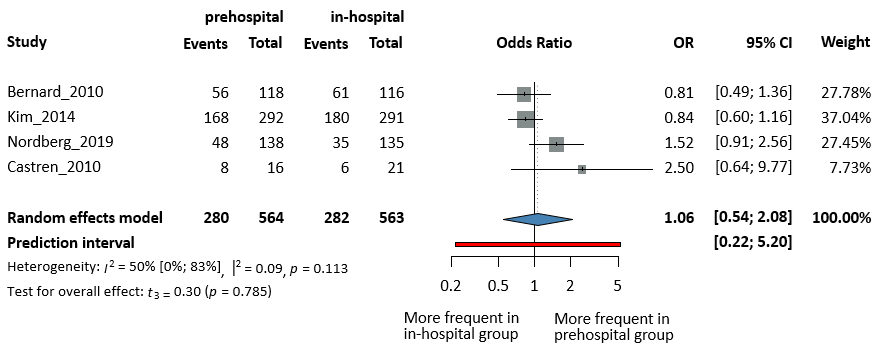
*Figure S1: Forest plot of patients with shockable rhythm exhibiting no significant difference in neurological outcomes. OR=odds ratio, CI=confidence interval.

*
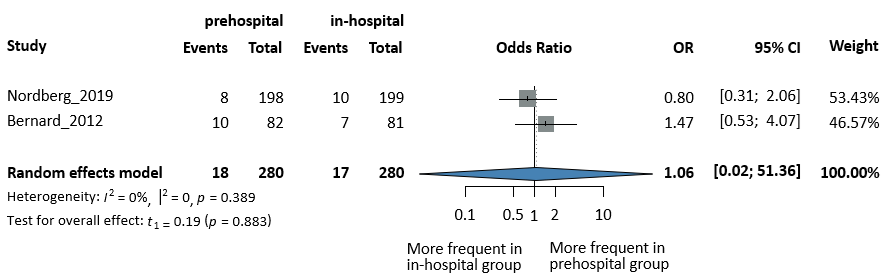
*Figure S2: Forest plot of patients with non-shockable rhythm exhibiting no significant difference in neurological outcomes. OR=odds ratio, CI=confidence interval.

*
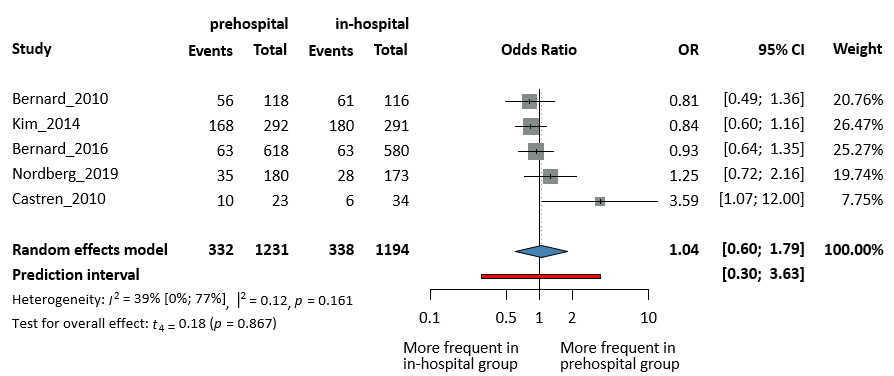
*

Figure S3: Forest plot of patients with ALS received within 10 minutes of collapse exhibiting no significant difference in neurological outcomes. OR=odds ratio, CI=confidence interval.

*
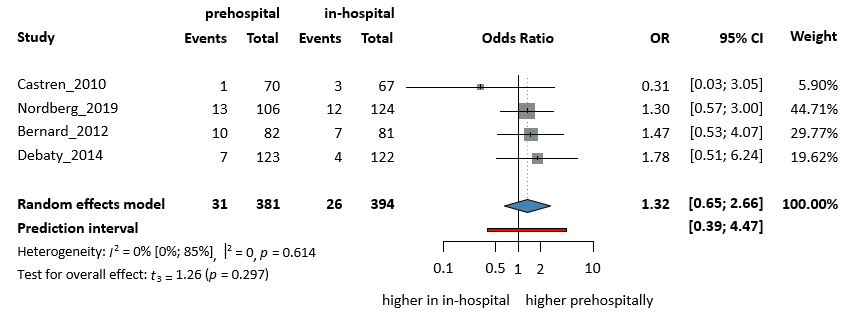
*Figure S4: Forest plot of patients with ALS received over 10 minutes of collapse exhibiting no significant difference in neurological outcomes. OR=odds ratio, CI=confidence interval.


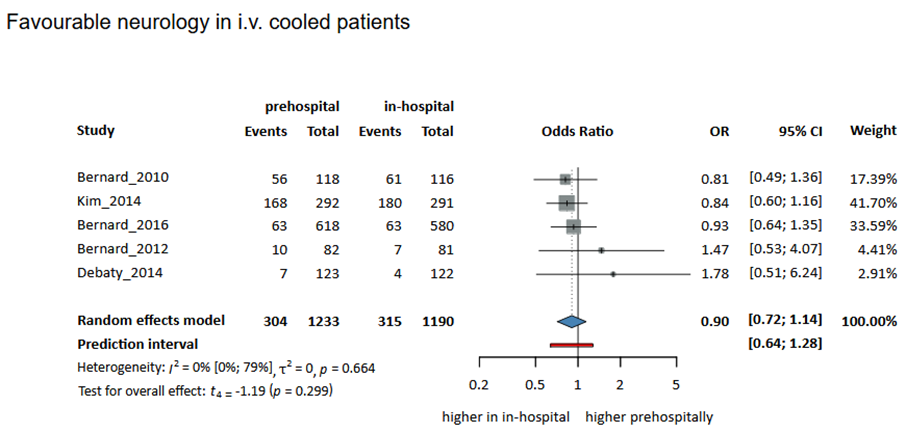


Figure S5: Forest plot of patients who received i.v. cooling exhibiting no significant difference in neurological outcomes. OR=odds ratio, CI=confidence interval.


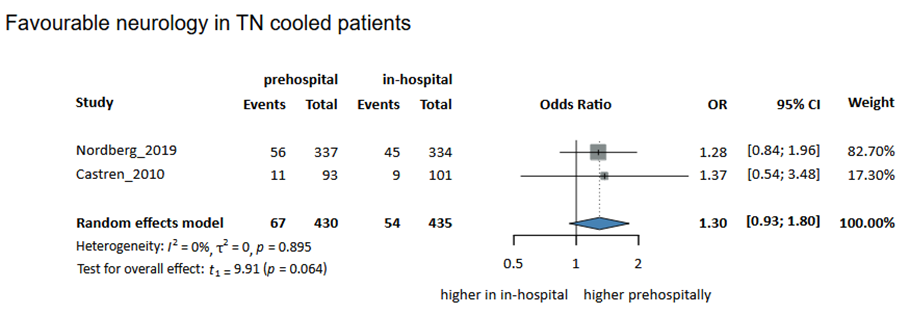


Figure S6: Forest plot of patients who received transnasal cooling exhibiting no significant difference in neurological outcomes. OR=odds ratio, CI=confidence interval.

*
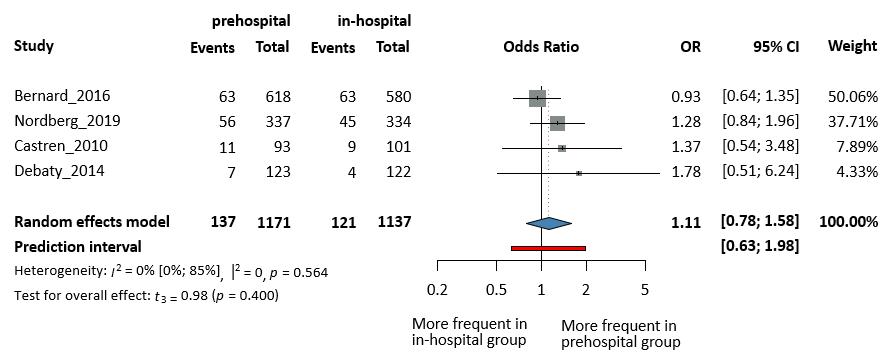
*Figure S7: Forest plot of patients who received intra-arrest cooling exhibiting no significant difference in neurological outcomes. OR=odds ratio, CI=confidence interval.

*
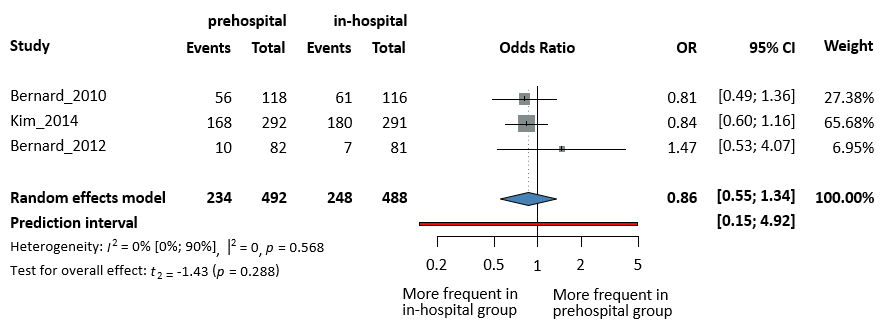
*Figure S8: Forest plot of patients who received post-ROSC cooling exhibiting no significant difference in neurological outcomes. OR=odds ratio, CI=confidence interval, ROSC=return of spontaneous circulation.

**SECONDARY OUTCOMES**

*
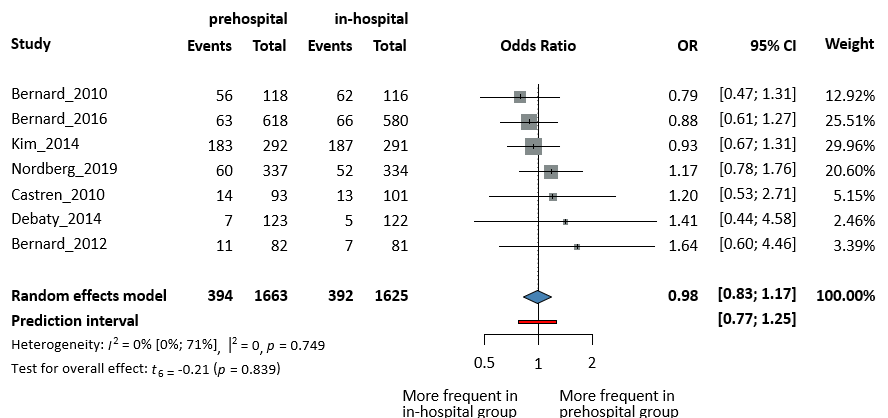
*

Figure S9: Forest plot of all patients exhibiting no significant difference in survival at hospital discharge*. *Nordbert et al. reported their results 90 days post cardiac arrest. OR=odds ratio, CI=confidence interval.

*
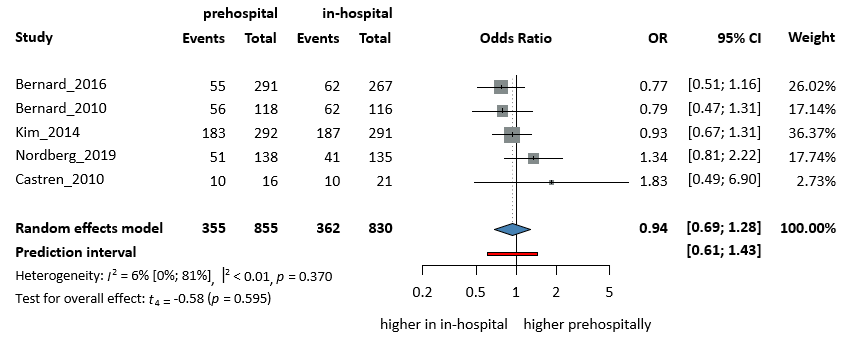
*

Figure S10: Forest plot of patients with shockable initial rhythm exhibiting no significant difference in survival at hospital discharge*. *Nordbert et al. reported their results 90 days post cardiac arrest. OR=odds ratio, CI=confidence interval.

*
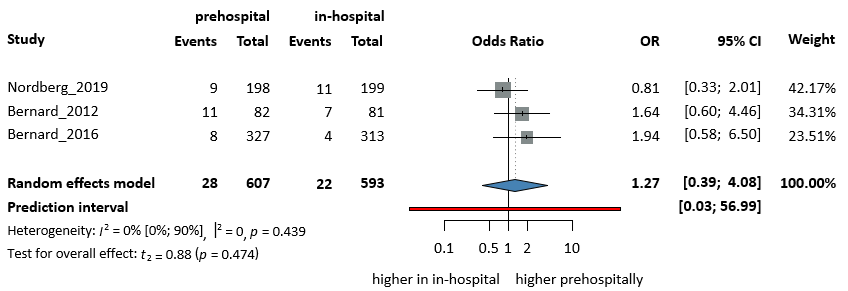
*

Figure S11: Forest plot of patients with non-shockable initial rhythm exhibiting no significant difference in survival at hospital discharge*. *Nordbert et al. reported their results 90 days post cardiac arrest. OR=odds ratio, CI=confidence interval.

**SAFETY OUTCOMES**

*
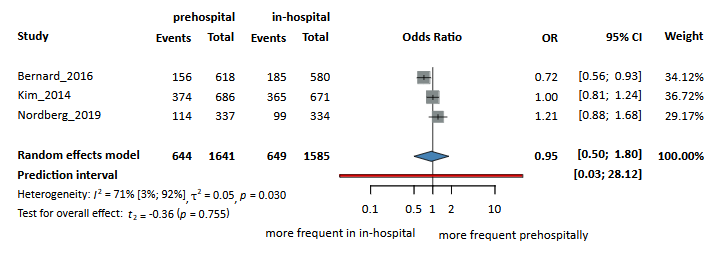
*Figure S12: Forest plot of patients exhibiting no significant difference in vasopressor need. OR=odds ratio, CI=confidence interval.

*
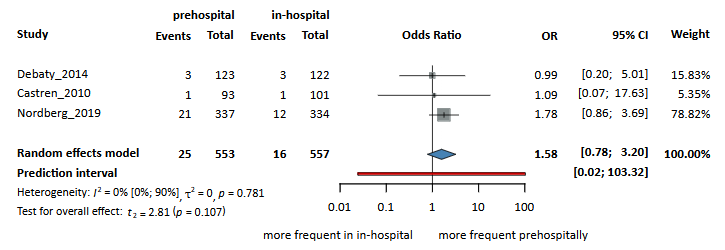
*Figure S13: Forest plot of patients exhibiting no significant difference in major bleeding. OR=odds ratio, CI=confidence interval.

*
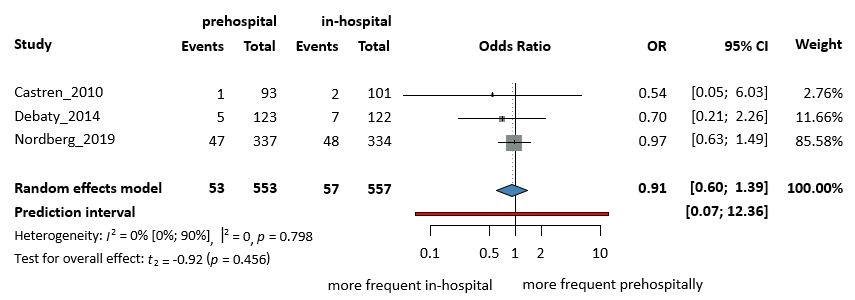
*

Figure S14: Forest plot of patients exhibiting no significant difference in arrhythmia. OR=odds ratio, CI=confidence interval.

**Results of The Risk of Bias and GRADE Assessment**

**RoB assessment on pre-defined outcomes.** The explanations in the figure legends are listed below each figure.


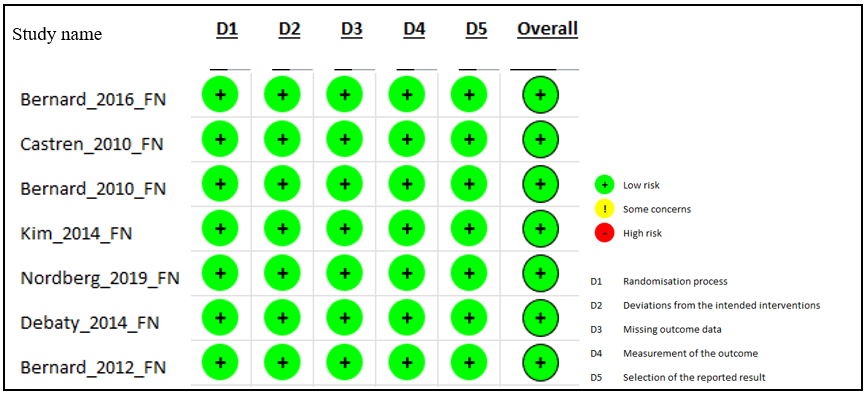


Figure S15: RoB assessment of **favourable neurology** (FN)


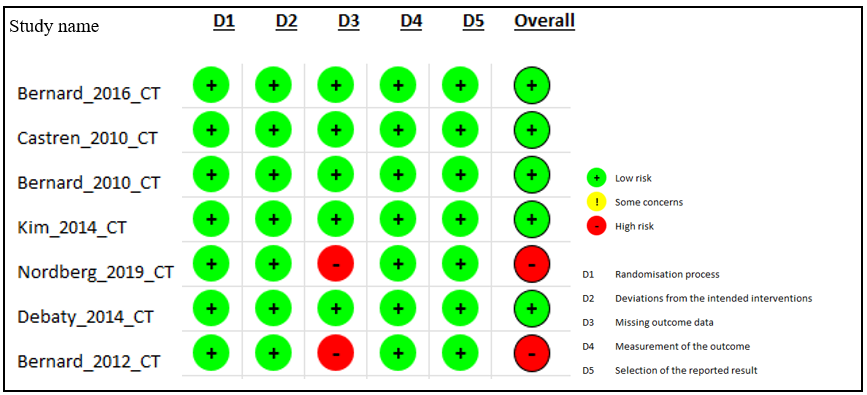
Figure S16: RoB assessment of **core temperature (CT) on hospital arrival**. In the study by Nordberg et al. (2019), core temperature data on admission were available for 90 of 149 patients in the intervention group and 73 of 142 patients in the control group. Bernard et al. (2012) reported missing admission core temperature data for 93 patients.


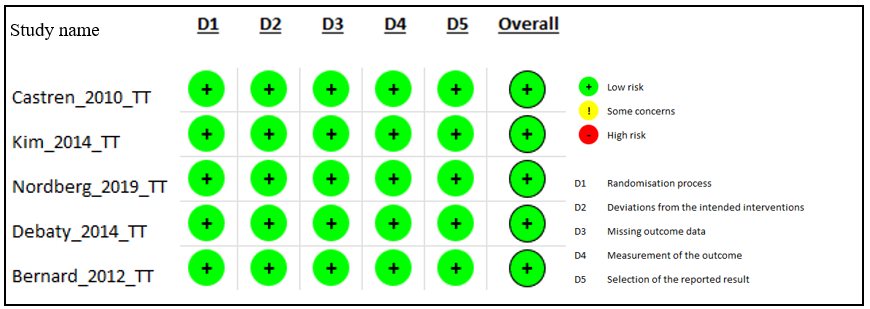
Figure S17: RoB assessment of **time to reach the target temperature (TT).**


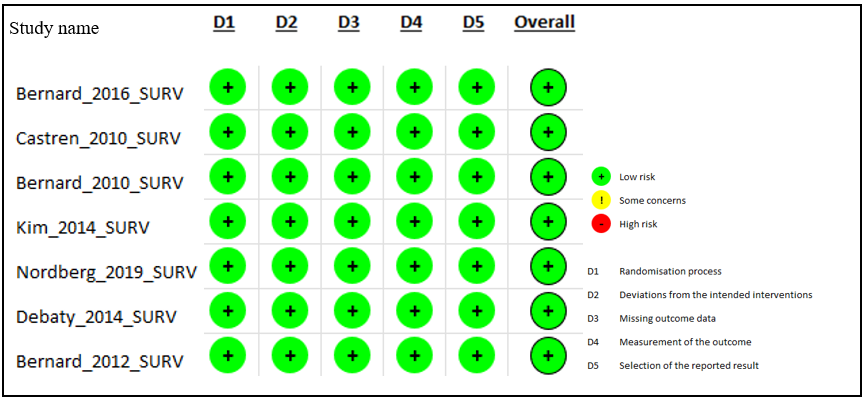
Figure S18: RoB assessment of **survival** (SURV).


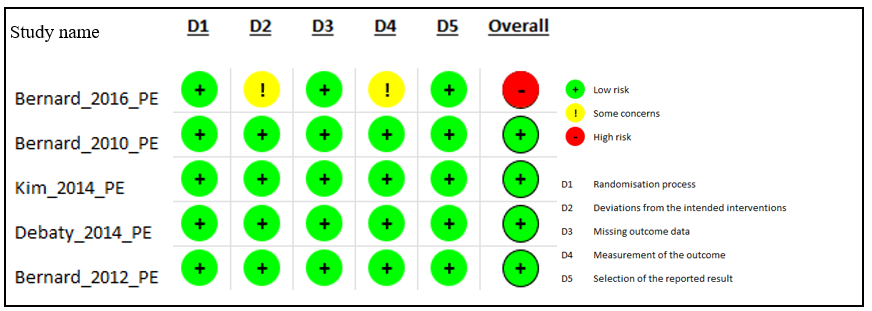
Figure S19. RoB assessment of **pulmonary oedema** (PE). 9 out of 580 people received cold fluids in the control group as deviation from intended interventions (D2) and the measurement of the outcome (D4) was not defined clearly, only “suspected” pulmonary oedema was reported.


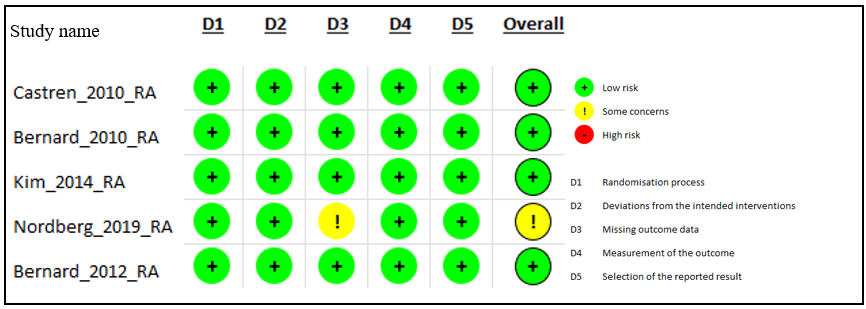
Figure S20: RoB assessment of **recurrent cardiac arrest** (RA). In the Nordberg 2019 article, concerning missing outcome data domain (D3), for recurrent arrest the data of 202 patients were available in the intervention group and the data of 185 patients were available in the control group, while the whole randomised was groups involved 337 and 334 people.


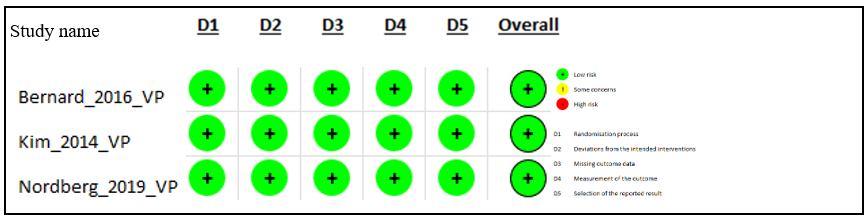
Figure S21: RoB assessment of **vasopressor need** (VP) of patients.


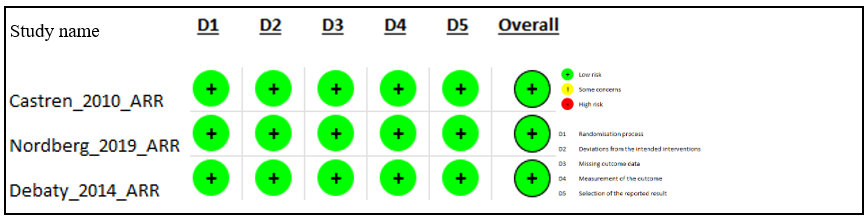
Figure S22: RoB assessment of **arrhythmia** (ARR) in patients.


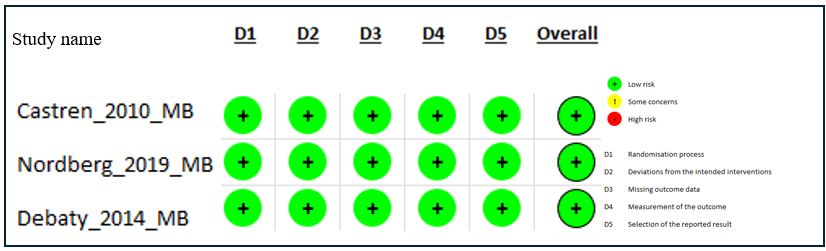
Figure S23: RoB assessment of **major bleeding** (MB) defined as intracranial, or fatal bleeding in patients.

**GRADE**

On the plot about the initial core temperature (Figure S16), the Nordberg 2019 article has high risk on the missing outcome domain (domain 3) due to a high number of patients missing the data of initial core T upon hospital arrival (data of 90/149 patients in the intervention group and 73/142 in the control group admitted were available). Also, Bernard et al. (2012) reported missing admission core temperature data for 93 patients. Considering these, the overall GRADE assessment on this outcome is “moderate” quality evidence.

On the plot about pulmonary oedema (Figure S19), in the Bernard 2016 article the “measurement of outcome” domain (domain 4) had high risk of bias because the assessment of pulmonary oedema was not defined clearly; it reported “suspected” pulmonary oedema with no pre-defined mode of measurement. Furthermore, in the same article, 9 people in the control group received cold i.v. fluids as faulty intervention (D2). Considering these, and also the fact that the timing of the assessment of pulmonary oedema is different in the articles (Bernard 2010, 2012 and Kim 2014 assesses pulmonary oedema developed during the way to the hospital, Debaty 2014 assesses it in the admitted patients in the first 72 hours, and Bernard 2016 does not specify the time of assessment), the overall GRADE assessment on this outcome is “moderate” quality evidence.

On the plot about recurrent arrest (Figure S20), in the Nordberg 2019 article the “missing outcome” domain (domain 3) had some concerns due to many patient data missing (202 patients had data out of 337 in the intervention group and 185 out of 334 in the control group). There was also a discrepancy in the time range of reporting about recurrent arrests. Bernard 2010 and 2012, Kim 2014 and Nordberg 2019 assess recurrent cardiac arrests developed during the way to the hospital, and Castren 2014 assesses it in the admitted patients in the first 7 days. Therefore, the overall GRADE assessment on this outcome is “moderate” quality evidence.

**GRADE table of all outcomes**


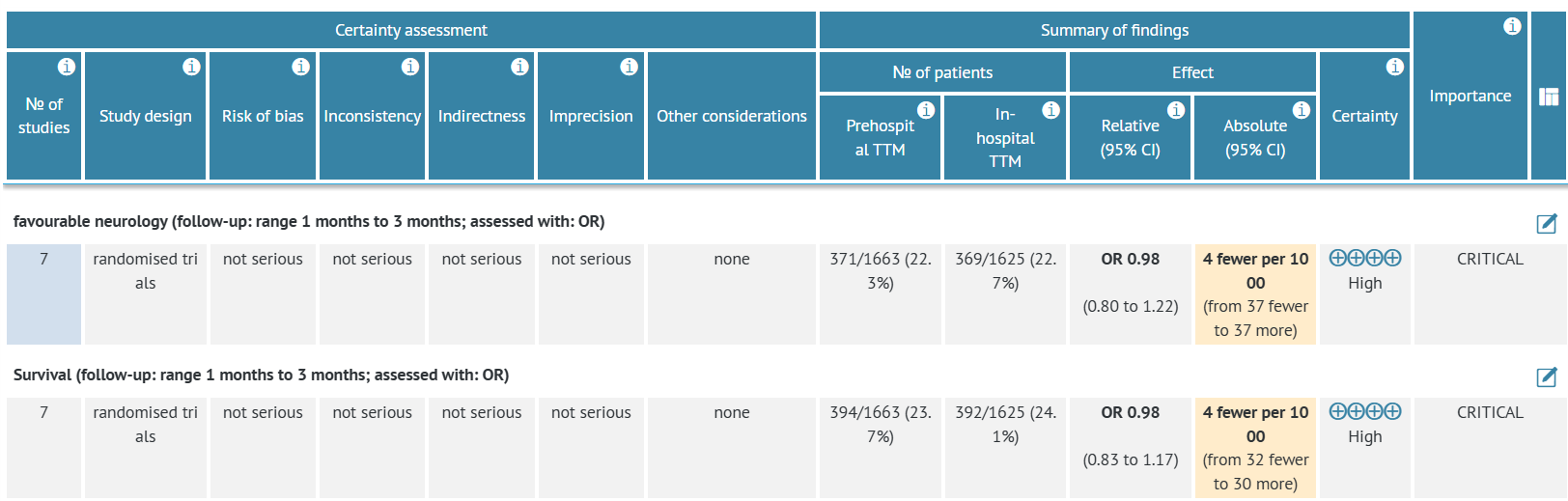


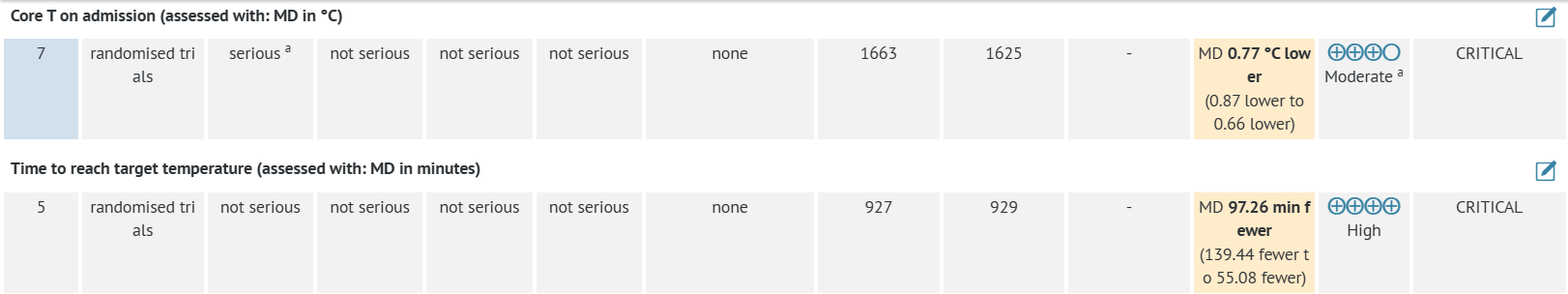


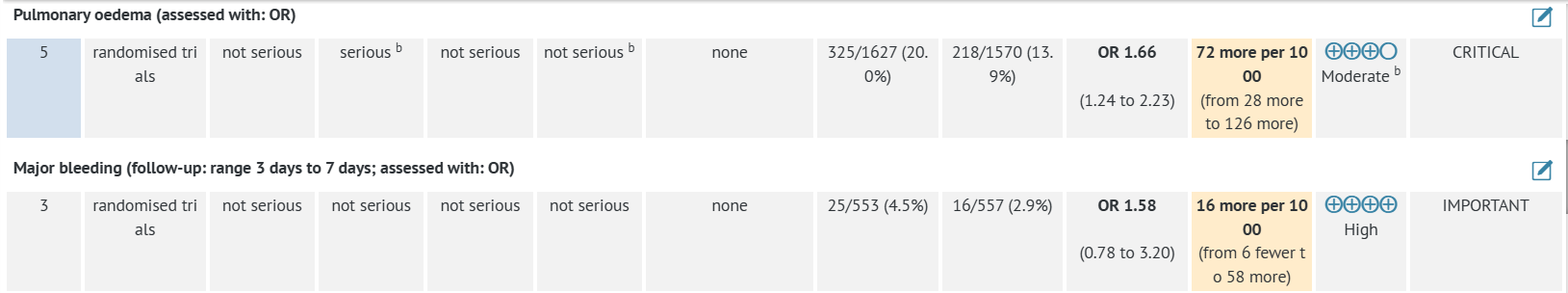


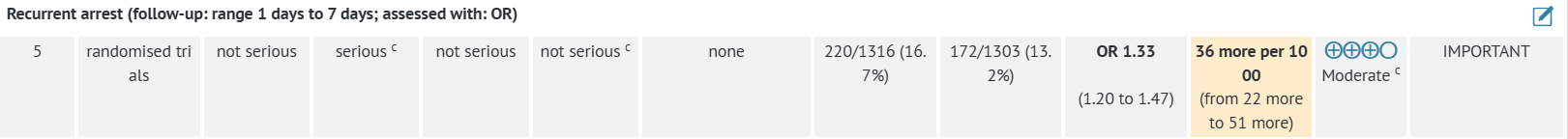


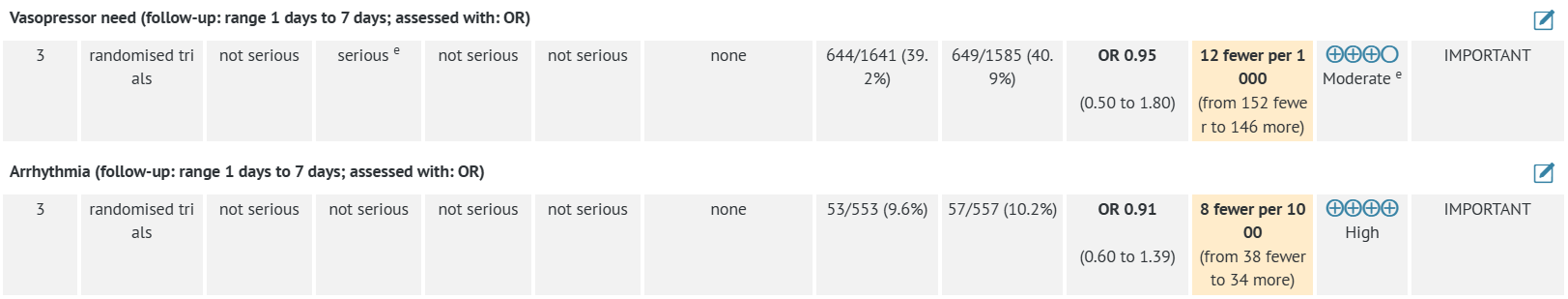


Table S4: GRADE table showing the certainty of evidence on a 4-rank scale (very low – low – moderate – high) of all included outcomes
